# Expression of *Bacillus subtilis* ABCF antibiotic resistance factor VmlR is regulated by RNA polymerase pausing, transcription attenuation, translation attenuation and (p)ppGpp

**DOI:** 10.1093/nar/gkac497

**Published:** 2022-06-14

**Authors:** Hiraku Takada, Zachary F Mandell, Helen Yakhnin, Anastasiya Glazyrina, Shinobu Chiba, Tatsuaki Kurata, Kelvin J Y Wu, Ben I C Tresco, Andrew G Myers, Gemma C Aktinson, Paul Babitzke, Vasili Hauryliuk

**Affiliations:** Faculty of Life Sciences, Kyoto Sangyo University and Institute for Protein Dynamics, Kamigamo, Motoyama, Kita-ku, Kyoto 603-8555, Japan; Department of Experimental Medical Science, Lund University, 221 00 Lund, Sweden; Department of Molecular Biology, Umeå University, Building 6K, 6L University Hospital Area, 90187 Umeå, Sweden; Department of Biochemistry and Molecular Biology, Center for RNA Molecular Biology, Pennsylvania State University, University Park, PA, USA; Department of Biochemistry and Molecular Biology, Center for RNA Molecular Biology, Pennsylvania State University, University Park, PA, USA; Department of Molecular Biology, Umeå University, Building 6K, 6L University Hospital Area, 90187 Umeå, Sweden; Faculty of Life Sciences, Kyoto Sangyo University and Institute for Protein Dynamics, Kamigamo, Motoyama, Kita-ku, Kyoto 603-8555, Japan; Department of Experimental Medical Science, Lund University, 221 00 Lund, Sweden; Department of Chemistry and Chemical Biology, Harvard University, Cambridge, MA, USA; Department of Chemistry and Chemical Biology, Harvard University, Cambridge, MA, USA; Department of Chemistry and Chemical Biology, Harvard University, Cambridge, MA, USA; Department of Experimental Medical Science, Lund University, 221 00 Lund, Sweden; Department of Biochemistry and Molecular Biology, Center for RNA Molecular Biology, Pennsylvania State University, University Park, PA, USA; Department of Experimental Medical Science, Lund University, 221 00 Lund, Sweden; Department of Molecular Biology, Umeå University, Building 6K, 6L University Hospital Area, 90187 Umeå, Sweden; University of Tartu, Institute of Technology, 50411, Tartu, Estonia

## Abstract

Since antibiotic resistance is often associated with a fitness cost, bacteria employ multi-layered regulatory mechanisms to ensure that expression of resistance factors is restricted to times of antibiotic challenge. In *Bacillus subtilis*, the chromosomally-encoded ABCF ATPase VmlR confers resistance to pleuromutilin, lincosamide and type A streptogramin translation inhibitors. Here we show that *vmlR* expression is regulated by translation attenuation and transcription attenuation mechanisms. Antibiotic-induced ribosome stalling during translation of an upstream open reading frame in the *vmlR* leader region prevents formation of an anti-antiterminator structure, leading to the formation of an antiterminator structure that prevents intrinsic termination. Thus, transcription in the presence of antibiotic induces *vmlR* expression. We also show that NusG-dependent RNA polymerase pausing in the *vmlR* leader prevents leaky expression in the absence of antibiotic. Furthermore, we demonstrate that induction of VmlR expression by compromised protein synthesis does not require the ability of VmlR to rescue the translational defect, as exemplified by constitutive induction of VmlR by ribosome assembly defects. Rather, the specificity of induction is determined by the antibiotic's ability to stall the ribosome on the regulatory open reading frame located within the *vmlR* leader. Finally, we demonstrate the involvement of (p)ppGpp-mediated signalling in antibiotic-induced VmlR expression.

## INTRODUCTION

Expression of genetic information involves coordinated regulation of transcription, translation and post-translational control of protein concentration and activity. The general transcription elongation factors NusA and NusG are global regulators of transcription in bacteria (1). In *Bacillus subtilis*, NusA and NusG stimulate transcription termination at weak intrinsic terminators containing hairpin mismatches and/or distal U tract interruptions (2). In addition, NusG-dependent RNA polymerase (RNAP) pausing occurs genome-wide via NusG interaction with conserved TTNTTT motifs in the nontemplate DNA strand within the paused transcription bubble (3–6). In addition to interacting with RNAP, *Escherichia coli* NusG interacts with ribosomal protein S10 of the lead ribosome, which physically couples transcription and translation in the so-called expressome complex (7–10); similar expressome architecture was observed in *Mycoplasma pneumoniae* (11). However, direct physical coupling of RNAP and the ribosome though NusG is not universal in bacteria, e.g. in *B. subtilis*, RNAP outpaces the lead ribosome through runaway transcription (12).

Secondary messenger nucleotides guanosine tetraphosphate (ppGpp) and pentaphosphate (pppGpp)—collectively referred to as (p)ppGpp—were initially discovered in *E. coli* as the key regulators of the transcriptional response to amino acid starvation (13). Decades of research have established (p)ppGpp as a near-universal pleotropic bacterial messenger, which regulates metabolism by targeting multiple key metabolic enzymes, and gene expression by regulating transcription, translation and ribosome assembly (14–18). By coordinating transcription and translation, (p)ppGpp plays a major role in adaptive responses to metabolic limitations (19), and it is possible that (p)ppGpp-mediated coordination of transcription and translation is equally important for bacterial adaptation to other stresses, such as antibiotic challenges. While the key role of (p)ppGpp in antibiotic tolerance and resistance is well recognised (20), the possible role of this alarmone in inducing expression of antibiotic resistance determinants has not been reported.

Antibiotics are small molecules that specifically inhibit essential cellular processes, such as protein synthesis. The ribosome is targeted by numerous antibiotic classes including pleuromutilins, lincosamides, streptogramins, phenicols and macrolides, and, conversely, bacteria employ an array of resistance mechanisms dedicated to protecting the ribosome from antibiotics (21,22). Expression of dedicated antibiotic resistance factors often involves tight regulation, since their constitutive production can be associated with a significant fitness cost (23). This dichotomy presents a regulatory challenge: while expression of the resistance factor needs to be increased upon antibiotic challenge, efficient expression of genetic information under these conditions is compromised by the drug. To induce the expression of resistance determinants in response to antibiotic challenges, bacteria often employ translation attenuation mechanisms that rely on the formation of alternative structures in the nascent RNA (24). Translational stalling on the nascent mRNA regulates transcription to induce the expression of several *B. subtilis* resistance factors: (i) the 23S rRNA methylase RlmAII which confers resistance to the macrolide tylosin (25), (ii) the multidrug resistance determinant BmrC/BmrD (26), a heterodimeric ATP-binding cassette (ABC) transporter (27) and (iii) the pleuromutilin, lincosamide and type A streptogramin (PLS_A_) resistance factor VmlR (28).

VmlR belongs to a family of ABCF ATPase antibiotic resistance (ARE) factors that have attracted significant attention in recent years (29–32). After being mis-annotated as efflux pumps for decades, these proteins are now recognised to operate on the ribosome by displacing antibiotics that target the peptidyltransferase center (PTC) and the nascent polypeptide exit tunnel (33–37). Horizontally transferred plasmid-encoded ARE ABCFs are exemplified by staphylococcal Vga (38) and PoxtA (39), as well as enterococcal OptrA (40). The rapid spread of these resistance factors is alarming, especially in the case of PoxtA and OptrA, which mediate resistance against last resort oxazolidinone antibiotics, such as linezolid (41). Some ARE ABCFs are chromosomally-encoded, with the best studied representatives found in Firmicutes, such as *Enterococcus faecalis* LsaA (37,42), *Listeria monocytogenes* VgaL/Lmo0919 (37,43), Staphylococcal Sal(A) (44) and *B. subtilis* VmlR (28,35). These proteins are responsible for intrinsic antibiotic resistance of these species. The genetic disruption of *vmlR* causes sensitivity to (i) the pleuromutilin tiamulin (35), (ii) lincosamides, such as the natural product lincomycin (28), as well as the more potent semi-synthetic antibiotic clindamycin and the recently developed, fully-synthetic iboxamycin (45) and (iii) the type A streptogramin virginiamycin M1 (28). The resistance effect is specific for these three antibiotic classes. For example, upon disruption of *vmlR* the sensitivity to the macrolides erythromycin and oleandomycin increases <2-fold, and there is no effect on the sensitivity to either type B streptogramin (S_B_) virginiamycin S (28) or to phenicols (35) (also see the summary MIC table, Table 1).

**Table 1.** Minimum inhibitory concentrations (MICs) of antibiotics against *B. subtilis* strains. 5 × 10^5^ CFU/mL (OD_600_ of approximately 0.0005) of *B. subtilis* liquid cultures were supplemented with increasing concentrations of antibiotics. After 16–20 h in the absence or presence of the antibiotic, bacterial growth was scored by eye. N.A., not available due to the selection markers used for strain construction rendering the strain phenicol resistant; N.D., not determined; PLS_A_ antibiotics that VmlR confers resistance to are highlighted in bold. MICs marked with an asterisk (*) were reported earlier by Brodiazhenko and colleagues (45). Lincomycin MICs for other mutant strains used in this work are summarised in Supplementary Table S1

	MIC, μg/ml			
Antibiotic	Wild-type 168	Δ*vmlR* (VHB5)	*vmlR-His6* (VHB223)	ppGpp^0^ (VHB63)
**Tiamulin**	80	0.3–0.6	80	20
**Retapamulin**	80	0.08	10	1.25
**Lincomycin**	80	2.5	20	5
**Iboxamycin**	2*	0.06*	0.5–1	0.25
**Virginiamycin M1**	>64	2	64	16
Linezolid	2	2	2	2
Florfenicol	3	3	3	3
Thiamphenicol	8	8	8	N.A.
Chloramphenicol	5	5	5	N.A.
Erythromycin	0.125	0.125	0.125	N.A.

VmlR expression is tightly controlled by the highly structured 5′ leader region of the *vmlR* transcript via a conditional transcription termination mechanism (28,46). In the absence of antibiotics, *vmlR* transcription terminates upstream of the *vmlR* open reading frame (ORF) at an intrinsic terminator that depends on both NusA and NusG for efficient termination (2,46,47). The addition of either virginiamycin M or lincomycin induces VmlR protein production by allowing transcription of the full-length mRNA (28,46).

In this study, we investigated the molecular mechanisms controlling antibiotic-inducible expression of VmlR. We have (i) clarified the connection between the inhibition of translation, induction of VmlR protein expression and the rescue of the translational defects by VmlR, (ii) revealed the role of NusG-dependent pausing in the regulation of VmlR expression, (iii) defined the transcription attenuation mechanism underlying the regulation of VmlR expression and (iv) uncovered the role of (p)ppGpp-mediated signalling in efficient induction of VmlR expression by antibiotic challenge.

## MATERIALS AND METHODS

### Bacterial strains, plasmids and oligonucleotides

Strains (and information regarding their sensitivity to lincomycin), plasmids, oligonucleotides, and synthetic DNA sequences used in this study are provided in Supplementary Table S1.

### Synthesis of iboxamycin

Iboxamycin was synthesised as described previously (48).

### Sequence and structure analyses

To retrieve homologous regions of the *B. subtilis* 5′ leader region, blastn (https://blast.ncbi.nlm.nih.gov/) was carried out using the NCBI representative genome database, using the upstream intergenic region of *vmlR* as the query. Sequences were visualised and aligned with Aliview v. 1.26 (49) and MAFFT v7.453 with the L-INS-i strategy (50). Secondary structures of the *vmlR* 5′ leader region were predicted using RNA Fold (51) and Mfold (52).

### Growth assays

*B. subtilis* strains were pre-grown on LB plates overnight at 30°C. Individual colonies were re-streaked on an LB plate, pre-grown at 37°C for approximately 4 h, and fresh colonies were used to inoculate 200 ml of LB (OD_600_ adjusted to 0.1) and growth was continued at 37°C. OD_600_ was monitored at half-hour intervals. For western blotting analysis, cells were collected at various times by centrifugation (8000 rpm, 5 min). After removing the supernatant, cell pellets were frozen by liquid nitrogen and stored at –80°C.

### Antibiotic sensitivity testing

Experiments were performed as described previously (35). Briefly, *B. subtilis* cells were pre-grown on LB plates supplemented with 1 mM IPTG if needed overnight at 30°C. Individual colonies were re-streaked on an LB plate, pre-grown at 37°C for approximately 4 h, and fresh colonies were used to inoculate filtered LB medium in the presence and absence of 1 mM IPTG, and OD_600_ adjusted to 0.01. The cultures were seeded on a 100-well honeycomb plate (Oy Growth Curves AB Ltd, Helsinki, Finland), and plates were incubated in a Bioscreen C (Labsystems, Helsinki, Finland) at 37°C with continuous shaking. The indicated concentration of antibiotics was added from the beginning or added after 90 min of growth (OD_600_ ≈ 0.1).

The minimum inhibitory concentrations (MIC) were calculated based on guidelines from the European Committee on Antimicrobial Susceptibility Testing (EUCAST) (http://www.eucast.org/ast_of_bacteria/mic_determination). *B. subtilis* strains were grown in LB medium supplemented with increasing concentrations of antibiotics after inoculation with 5 × 10^5^ CFU/ml (OD_600_ of ∼0.0005). After 16–20 h at 37°C without shaking, the presence or absence of bacterial growth was scored by eye.

### Antibiotics treatment and sampling for western blotting analysis

*B. subtilis* strains were pre-grown on LB plates overnight at 30°C. Individual colonies were streaked on an LB plate, incubated at 37°C for ∼4 h, and fresh colonies were used to inoculate LB culture (OD_600_ adjusted to 0.02) that were grown at 37°C. At OD_600_ of ≈0.25, the indicated concentration of antibiotics was added. After the indicated time, treated cells were collected by centrifugation (8000 rpm, 5 min). After removing the supernatant, cell pellets were frozen by liquid nitrogen and stored at –80°C.

### Preparation of *B. subtilis* 30S ribosomal subunits

*B. subtilis* 70S ribosomes were purified as described previously (53), diluted with low-magnesium HEPES:Polymix buffer (1 mM MgOAc) and incubated on ice for 30 min to promote the dissociation of subunits. The sample was then resolved on a 10–40% sucrose gradient in overlay buffer (60 mM NH_4_Cl, 1 mM Mg(OAc)_2_, 0.25 mM EDTA, 3 mM β-mercaptoethanol, 20 mM Tris–HCl (pH 7.5) in a zonal rotor (Ti 15, Beckman, 18 h at 21 000 rpm). The peak containing pure 30S ribosomal subunits was pelleted by centrifugation (48 h at 35 000 rpm), and the final ribosomal preparation was dissolved in HEPES:Polymix buffer with 5 mM Mg(OAc)_2_.

### Sucrose gradient fractionation before and after cold stress

The experiments were performed as described previously (53) with minor modifications. *B. subtilis* strains were pre-grown on LB plates overnight at 30°C. Individual colonies were re-streaked on LB plate, grown at 37°C for ∼4 h, and fresh colonies were used to inoculate 200 mL LB cultures that were grown at 37°C. At OD_600_ of 0.2, the cultures were transferred to 20°C, grown for an additional 60 min, and then samples were collected by centrifugation. After removing the supernatant, cell pellets were frozen by liquid nitrogen and stored at -80°C. Cell pellets were dissolved in 0.5 mL of HEPES:Polymix buffer (5 mM Mg(OAc)_2_) supplemented with 1 mM PMSF, lysed using a FastPrep homogenizer (MP Biomedicals) by four 20 s pulses at a speed of 4.0 m/s with chilling on ice for 3 min between each cycle), and clarified by centrifugation. These samples were also used for western blotting analysis as described below. Ten *A*_260_ units of each extract were loaded onto 10–35% (w/v) sucrose density gradients in Polymix buffer, 5 mM Mg(OAc)_2_. Gradients were resolved by centrifugation at 36 000 rpm for 3 h at 4°C in a SW41 rotor (Beckman). Sucrose gradients were both poured and fractionated using a Biocomp Gradient Station (BioComp Instruments).

### Western blotting analysis

Frozen cell pellets were treated as described above for sucrose gradient fractionation. 0.2 *A*_260_ unit of each cell extract was boiled with 2× SDS loading buffer (100 mM Tris–HCl pH 6.8, 4% SDS (w/v) 0.02% Bromophenol blue, 20% glycerol (w/v) 4% β -mercaptoethanol) were resolved by 10% or 12% SDS PAGE and transferred to a nitrocellulose membrane (Trans-Blot Turbo Midi Nitrocellulose Transfer Pack, Bio-Rad, 0.2 μm pore size) with the use of a Trans-Blot Turbo Transfer Starter System (Bio-Rad) (10 min, 2.5 A, 25 V). C-terminally His_6_-tagged VmlR and BmrD were detected using anti-His_6_ primary antibodies (Wako, 010-21861; 1:10 000 dilution) combined with anti-mouse-HRP secondary antibodies (Rockland; 610-103-040; 1:10 000 dilution). 50S ribosomal proteins L3 and L11 were detected using anti-L3 and anti-L11 primary antibodies (1:20 000 dilution, both provided by Fujio Kawamura), combined with goat anti-rabbit IgG-HRP secondary antibodies (Sigma-Aldrich, A0545; 1:10 000 dilution). The hibernation Promoting Factor (HPF) was detected using a polyclonal anti-HPF antibody (1:10 000 dilution, provided by Fujio Kawamura (54)) combined with goat anti-rabbit IgG-HRP secondary antibodies (Sigma-Aldrich, A0545; 1:10 000 dilution). ECL detection was performed using WesternBright™ Quantum (K-12042-D10, Advansta) western blotting substrate and an ImageQuant LAS 4000 (GE Healthcare) imaging system.

### Functional genomics analyses of NusA-stimulated termination and NusG-dependent pause sites

We previously performed native elongating transcript sequencing followed by RNase I digestion (RNET-seq) under wild-type (PLBS730, +IPTG), NusA depletion (PLBS730, –IPTG), and Δ*nusG* (PLBS731, +IPTG) conditions (3,55). The RNET-seq data are available in the National Center for Biotechnology Information Sequence Read Archive (BioProject ID numbers PRJNA603835) and GEO (accession number GSE186285). Term-seq was previously performed under wild-type, NusA depletion, *nusG* deletion, and combined NusA depletion / *nusG* deletion conditions (47), and the data are available in GEO (accession numbers GSE67058 and GSE154522).

### *In vitro* transcription

We designed a gBlock gene fragment that contained modifications of the natural *vmlR* promoter region, resulting in consensus –35 and extended –10 promoter elements, as well as ideal spacing between the –10 element and the +1 nucleotide. The final promoter sequence was **TTGACA**TGAATTTAAAGGTA**TGTTATAAT**GTTTGT**A**, where bold nucleotides highlight the –35 and extended -10 elements, and the +1 nucleotide. We also inserted two adenosine residues five nucleotides downstream of +1 to yield a 14-nt G-less cassette. The G-less cassette was followed by the *vmlR* leader region and the first 18 codons of the *vmlR* coding sequence. After the gBlock was amplified by PCR using primers vmlR-amp-For and vmlR-amp-Rev, the PCR product was cloned into the *Eco*RI and *Hin*dIII sites of pTZ19R (Thermo Fisher Scientific), resulting in plasmid pZM72. The NusG-dependent pause motif in the *vmlR* leader (3) was subsequently mutated from TTATTT to TTACAA via the QuikChange protocol (Agilent) using pZM72 as template and primers vmlR-mut-For and vmlR-mut-Rev, resulting in plasmid pZM73. Templates for *in vitro* transcription containing the WT or mutant NusG-dependent pause motif were PCR-amplified using pZM72 and pZM73 as templates and the primer pair vmlR-amp-For and vmlR-amp-Rev, respectively.

Analysis of RNAP pausing and termination was performed as described previously with modifications (2). Halted elongation complexes containing a 14-nt transcript were formed for 5 min at 37°C by combining equal volumes of 2x template (50–200 nM) with 2× halted elongation complex master mix containing 80 μM ATP and CTP, 2 μM UTP, 100 μg/ml bovine serum albumin, 150 μg/ml (0.38 μM) *B. subtilis* RNAP holoenzyme, 0.76 μM σ^A^, 2 μCi of [α-^32^P]UTP and 2× transcription buffer (1× = 40 mM Tris–HCl, pH 8.0, 5 mM MgCl_2_, 5% trehalose, 0.1 mM EDTA and 4 mM dithiothreitol). RNAP and σ^A^ were added from a 10× stock solution containing 0.75 mg/ml RNAP and 0.175 mg/ml σ^A^ in enzyme dilution buffer (20 mM Tris–HCl, pH 8.0, 40 mM KCl, 1 mM dithiothreitol and 50% glycerol). A 4× solution containing either 0 or 4 μM of NusG and/or NusA in 1x transcription buffer were added and the resulting solution was incubated for 5 min at 23°C. A 4× extension master mix containing 80 μM KCl, 600 μM of each NTP, 400 μM rifampicin, in 1× transcription buffer was added. For each pausing assay, the reaction was incubated at 23°C, with aliquots removed and added to 2× stop/gel loading solution (40 mM Tris-base, 20 mM Na_2_EDTA, 0.2% sodium dodecyl sulfate, 0.05% bromophenol blue, and 0.05% xylene cyanol in formamide) at the specified time points. A 30 min time point was included for all pausing assays, which we considered to be our termination condition. RNA bands were separated on standard 5% sequencing polyacrylamide gels, which were subsequently imaged on a Typhoon 8600 Phosphorimager (GE Healthcare Life Sciences). Each *in vitro* transcription experiment was repeated at least twice, with representative gels shown. Termination efficiencies and pausing half-lives were quantified as described previously (56).

### Toeprint assay

Toeprint assays followed a published procedure (25). DNA templates for *in vitro* transcription were generated by PCR using plasmids pYH389 (WT) and pYH399 (SD mutant) and primer pairs T7-WT-For/PCR-Rev and T7-mut-For/PCR-Rev, respectively. WT and SD mutant *vmlR* RNA were synthesized using the RNAMaxx kit (Agilent Technologies). RNA containing from +1 to +165 relative to the *vmlR* transcription start site was gel purified. The toeprint primer (vmlR-Toeprint) complementary to +79 to +101 of *vmlR* was 5′-end labelled using T4 polynucleotide kinase (New England Biolabs) and [γ-^32^P] ATP (7000 Ci/mmol) (Perkin Elmer). Each toeprint reaction mixture (10 μl) contained 2 μl of hybridization mixture (1 μl of 1 μM labelled primer and 1 μl of 1 μM RNA), 2 μl of 17 μM *B. subtilis* 30S ribosomal subunits or dilution buffer, 6 μl of reaction mixture (1× reverse transcriptase buffer, 10 μM *Escherichia coli* fMet-tRNA_i_^fMet^, 2 μg of yeast RNA, 375 μM each deoxynucleoside triphosphate (dNTP), 10 mM dithiothreitol and 0.1 mg/ml bovine serum albumin). Hybridization mixtures were incubated for 3 min at 80°C followed by slow cooling for 10 min at room temperature. Prior to addition, 30S ribosomal subunits were activated by incubation for 15 min at 37°C. After addition, 30S ribosomal subunit mixtures were incubated for 10 min at 37°C. 1 μl AMV (1 U) was added, and incubation was continued for 15 min at 37°C. Reactions were stopped by the addition of 10 μl of gel loading buffer (95% formamide, 0.025% SDS, 20 mM EDTA, 0.025% bromophenol blue, 0.025% xylene cyanol). Samples were heated at 95°C for 5 min and then fractionated through standard 6% polyacrylamide/8 M urea sequencing gels, which were subsequently imaged on a Typhoon 8600 Phosphorimager.

## RESULTS AND DISCUSSION

### The *vmlR* leader region contains several RNA structures, a predicted upstream ORF (uORF), RNAP pause signals, and an intrinsic terminator

**Figure 1.**
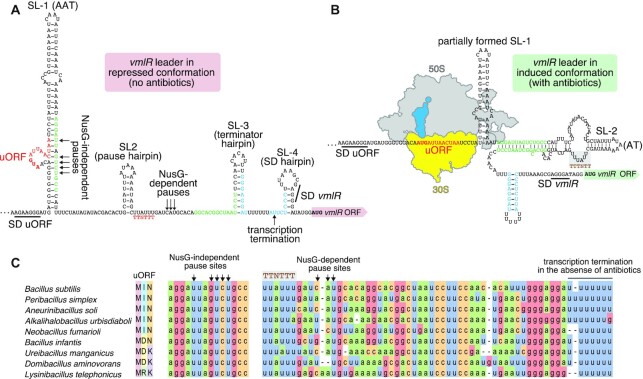
Predicted repressed and induced conformations of the *B. subtilis vmlR* leader region. (**A**) Secondary structure of the repressed conformation of the 219 nt-long mRNA region upstream of the *vmlR* start codon as predicted by the RNA Fold Web Server (51). Shine-Dalgarno (SD) sequences for the uORF and *vmlR* are shown. The four predicted stem loops (SL-1 anti-antiterminator [AAT] hairpin, SL-2 pause hairpin, SL-3 intrinsic terminator hairpin, SL-4 *vmlR* SD-sequestering hairpin) are labelled. NusG-independent and NusG-dependent pause sites determined by RNET-seq are marked with black arrows. The uORF located within SL-1 and the NusG TTNTTT pause motif are in red. The transcription attenuation termination site is indicated with an arrow. Residues shown in green may base pair and form part of an alternative antiterminator (AT) structure, which would be expected to occur if lincomycin stalls translation of the uORF. Base-pairing of the blue residues in readthrough transcripts would disrupt the *vmlR* SD-sequestering hairpin to allow more efficient translation initiation. (**B**) Secondary structure of the *vmlR* mRNA 5′ leader in the induced conformation. This conformation is incompatible with formation of the SL-1, SL-3 and SL-4 hairpins, thus allowing transcription of the full-length *vmlR* mRNA and its efficient translation, respectively. (**C**) Sequence conservation of the *vmlR* leader region in species of *Bacilli*. The full-length 5′ *vmlR* leader alignment is shown in Supplementary Figure S1.

RNA structural prediction of the *vmlR* 5′ leader region using RNAfold (51) and Mfold (52) revealed four stem-loops, SL-1 to SL-4 (Figure 1A). The first stem-loop, SL-1, contains a putative 5′ uORF encoding the Met-Ile-Asn tripeptide, uORF(MIN) (46) (Figure 1C, Supplementary Figure S1). The predicted Shine-Dalgarno (SD) sequence that could be capable of driving translation of the uORF(MIN) is located in a single-stranded region preceding SL-1. The uORF itself is encoded in a bulge region within SL-1. Several previously identified NusG-independent pause sites are located across from uORF within SL-1 (3). The second structure, SL-2, is the putative pause hairpin for a cluster of three previously identified NusG-dependent pauses (3), while SL-3 is the terminator hairpin of a previously identified NusA/NusG-stimulated intrinsic terminator (2,47). This terminator could be responsible for the premature transcription termination in the absence of ‘cognate’ antibiotics. The final stem-loop, SL-4, partially sequesters the *vmlR* SD sequence. While most transcripts would likely terminate before SL-4 is reached under non-inducing conditions, SL-4 could potentially serve as an additional regulatory layer that inhibits VmlR synthesis from mRNA transcripts that fail to terminate in the leader region.

Transcription attenuation typically relies on overlapping (mutually exclusive) antiterminator (AT) and intrinsic terminator hairpin structures that can form in the nascent transcript (57,58). Since the AT is encoded upstream of the terminator hairpin, the AT has the opportunity to form first. In some cases, a third RNA structure called an anti-antiterminator (AAT) participates in attenuation. The AAT precedes and overlaps the AT structure. Thus, formation of the AAT favours termination by preventing formation of the AT. We have formulated an initial model of transcription attenuation-based regulatory mechanism for inducible *vmlR* expression (Figure 1A,B). In this model SL-1 serves as an AAT, thereby promoting termination in the *vmlR* leader just downstream of the terminator hairpin (SL-3). In addition, NusG-independent RNAP pausing would provide sufficient time for translation of the uORF(MIN), and in the presence of a ‘cognate’ antibiotic (such as lincomycin that VmlR protects from), but not ‘non-cognate’ antibiotics (such as erythromycin that VmlR does not protect from), would induce ribosomal stalling on the uORF. Ribosomal stalling in this region would prevent formation of the base of SL-1. In this situation a predicted AT structure could form with SL-2 at its apex, which would promote transcription readthrough into the *vmlR* coding sequence. Moreover, formation of the AT would simultaneously promote formation of a structure that could compete with SL-4, thereby alleviating repression of *vmlR* translation, leading to efficient synthesis of the antibiotic resistance factor. Lastly, NusG-dependent pausing could provide sufficient time for AAT formation in the absence of translational stalling, increase the time for translational stalling in the presence of a cognate antibiotic, or both.

### VmlR expression is efficiently induced only by VmlR-cognate antibiotics

To begin testing our model, we modified the chromosomally-encoded *vmlR* gene in the *B. subtilis* wild-type strain 168 by C-terminally extending the protein with a His_6_ tag, yielding the stain VHB223 (*trpC2 vmlR-His_6_*), and thus allowing specific detection of the VmlR-His_6_ protein by immunoblotting with C-terminus-specific anti-His antibodies. While, as judged by antibiotic sensitivity, His_6_-tagging did not abrogate VmlR’s functionality, the protective activity decreased for most of the antibiotics tested (Table 1). The *vmlR-His_6_* strain (VHB223) exhibited wild-type levels of resistance to pleuromutilin tiamulin. VmlR-His_6_ also conferred resistance to the PTC-targeting PLS_A_ antibiotics: pleuromutilin retapamulin (which, as we show here, VmlR provides resistance against, see Table 1), lincosamides lincomycin and iboxamycin, and type A streptogramin virginiamycin M1, although the strain is 8-, 4-, 2-to-4- and 2-fold more sensitive than the parental wild-type strain, respectively. We detected no differences in sensitivity of the *vmlR-His_6_* strain to phenicols chloramphenicol and thiamphenicol, oxazolidinone linezolid or macrolide erythromycin as compared to the wild type or Δ*vmlR* (VHB5) strains. As we have shown before (35), the C-terminal extension (CTE) of VmlR contacts with the small subunit of the ribosome, and deletion of the CTE renders the protein unable to confer resistance. While this information helps rationalise the slight deleterious effect of C-terminal His_6_-tagging, it does not explain why resistance is differentially affected for different antibiotics: interaction of the CTE with the cleft on the 30S subunit formed by the proteins uS7 and uS11 is located far from the antibiotic binding sites on the 50S.

**Figure 2.**
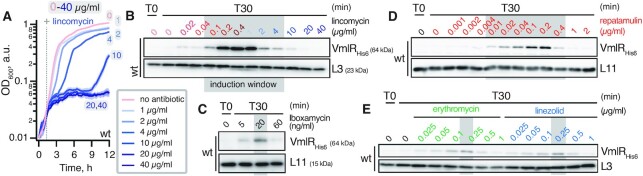
Efficiency of antibiotic-dependent induction of VmlR expression is correlated with the resistance spectrum of the ABCF. (**A**) Growth of wild-type *B. subtilis* 168 (VHB223) treated with 0–40 μg/ml of lincomycin. Analogous experiments with low lincomycin concentrations that do not affect the bacterial growth (0–0.4 μg/ml) are shown in Supplementary Figure S2A. (**B**–**E**) Increasing concentrations of antibiotics were added after bacterial cultures reached OD_600_ of ≈0.2 (T0) and expression of VmlR_His6_ was assessed by α-His_6_ immunoblotting after 30 min of antibiotic treatment (T30). VmlR confers resistance to lincomycin, iboxamycin and retapamulin, but not to erythromycin and linezolid (Table 1). Since liquid media growth experiments were performed in plate format using the Bioscreen C system, the OD_600_ is presented in arbitrary units (a.u.); the standard deviation of three biological replicates is indicated with pale shading. The final concentrations of antibiotics are indicated on the panels. All experiments were performed at least twice yielding similar results.

To study antibiotic-induced VmlR expression, we treated the wild-type strain for 30 min with increasing antibiotic concentrations. We selected three VmlR-cognate antibiotics: lincomycin (a known inducer of VmlR expression (28,46)), iboxamycin and retapamulin. We also selected two non-cognate antibiotics that VmlR does not provide resistance to: linezolid and erythromycin. The lincomycin treatment resulted in strong induction of the α-VmlR-His_6_ signal, with the maximum VmlR protein levels – ‘the induction window’ – observed in the presence of 0.2–1 μg/ml of antibiotic (Figure 2A, B). At these concentrations, bacterial growth was virtually unaffected (Supplementary Figure S2A). The decrease in VmlR levels in the presence of lincomycin at concentrations higher than 1 μg/ml may be due to the antibiotic causing global inhibition of protein synthesis as suggested by the pronounced inhibition of growth (Figure 2A). While expression of VmlR was also induced by iboxamycin, the induction is weaker (Figure 2C), possibly reflecting much more efficient inhibition of bacterial growth (and protein synthesis) by this fully synthetic drug (40-fold lower MIC as compared to lincomycin, see Table 1). Furthermore, VmlR expression is efficiently induced by retapamulin (Figure 2D). Finally, while we also detected some induction of VmlR expression in the presence of the non-cognate antibiotics erythromycin and linezolid, the induction is substantially weaker than in the case of the cognate antibiotics (Figure 2E).

### VmlR is induced by defects in translation and induction does not depend on VmlR activity

A recent study of Staphylococcal antibiotic resistance factor Vga(A) suggested that the resistance profile of this ABCF factor is the key determinant of which antibiotics induce its expression (59). To establish whether the same holds true for VmlR, we examined the possible connection between the ability of VmlR to counteract the translation defect caused by the antibiotic by providing resistance and the ability of the translational defect to induce VmlR expression.

The lack of efficient VmlR induction by erythromycin and linezolid could be due to the regulatory elements controlling VmlR expression failing to sense and respond to translational defects caused by non-cognate antibiotics. The two tested VmlR-inducing PLS_A_ antibiotics retapamulin and lincomycin are expected to stall the ribosome at initiation. Initiation-specific stalling was directly shown for retapamulin (60). Biochemical and structural studies demonstrated that lincosamides lincomycin (61,62) and iboxamycin (63) interfere with efficient accommodation of the A-site tRNA, and, therefore, in the cellular context are expected to compromise the efficient progression from initiation to polypeptide elongation. On the other hand, linezolid and erythromycin target elongation; efficient stalling by these antibiotics is context-specific and relies on the interactions between the antibiotic, the nascent polypeptide chain, and the ribosome peptide exit tunnel (64–70). If the predicted three-codon uORF(MIN) (46) plays a role in controlling VmlR expression, it is possible that lincosamides and pleuromutilins would cause the ribosome to stall leading to VmlR induction, whereas ribosomes would fail to stall in the presence of oxazolidinones or macrolides, and therefore would not induce VmlR expression. Another explanation for inefficient VmlR induction when the cells are challenged with antibiotics that VmlR does not confer resistance to is that while inhibition of ribosomal function is necessary and sufficient to induce VmlR expression, due to the lack of protection from these non-cognate antibiotics, the net increase in VmlR expression is negligible because the protein synthesis is effectively abrogated. Thus, the width of the antibiotic concentration window in which VmlR expression is induced, but global protein production is not yet suppressed, reflects the difference in antibiotic sensitivity prior to VmlR induction and post-induction. This induction window is expected to be relatively wide for cognate antibiotics and narrow for non-cognate antibiotics. Finally, one would expect the window to be narrower in the case of more potent antibiotic variants, such as iboxamycin (compare Figure 2B and Figure 2C).

**Figure 3.**
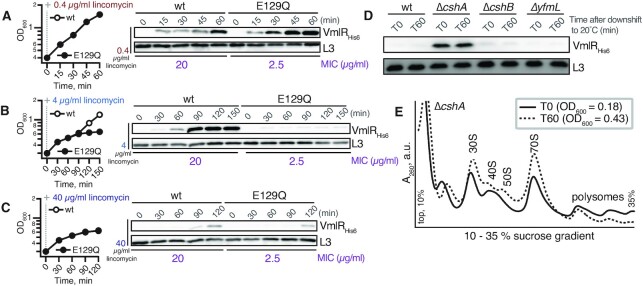
VmlR is induced by defects in translation and induction does not depend on VmlR activity. (A–C) Lincomycin was added at a final concentration of 0.4 μg/ml after wild-type (VHB627) or the ATPase-deficient E129Q mutant VmlR (VHB628) strains reached OD_600_ of 0.2–0.3. (**A**) Permissive concentration (0.4 μg/ml) for both strains; the two growth curves are identical and overlay each other. (**B**) Lincomycin was added at a final concentration of 4 μg/ml, which is a subinhibitory concentration for the wild-type strain and an inhibitory concentration for the *vmlR^E129Q^* strain. (**C**) Lincomycin was added at a final concentration of 40 μg/ml, which is an inhibitory concentration for both strains. Efficient induction of VmlR expression was conditional on bacterial growth, but not on VmlR functionality since the E129Q variant does not confer resistance to lincomycin. VmlR_His6_ expression was detected by α-His_6_ immunoblotting. (**D**) The effect of RNA helicase mutations on induction of VmlR expression. Wild-type 168 (VHB223) and isogenic Δ*cshA* (VHB224), Δ*cshB* (VHB225) and Δ*yfmL* (VHB226) strains were grown to an OD_600_ of ≈0.2 (T0) at which time the cultures were transferred to 20°C, and then grown for an additional 60 min (T60). VmlR_His6_ expression was detected by α-His_6_ immunoblotting. (**E**) Polysome profile of the Δ*cshA* strain before and after temperature downshift from 37°C to 20°C. Analogous experiments with wild-type, Δ*cshB* and Δ*yfmL* strains are shown in Supplementary Figure S3. All experiments were performed at least twice, yielding similar results.

To test this induction window hypothesis, we constructed a *vmlR^E129Q^* (VHB628) strain in which the first ATPase cassette of VmlR is rendered inactive by substituting the catalytic glutamic acid residue of the Walker B motif for glutamine. This substitution rendered the ABCF resistance factor non-functional, resulting in the *vmlR^E129Q^* strain being as sensitive to lincomycin as the Δ*vmlR* strain (Supplementary Table S1). When challenged with 0.4 μg/ml lincomycin, the wild-type and *vmlR^E129Q^* strains did not exhibit a growth defect and both induced expression of either wild-type or inactive EQ-substituted VmlR (Figure 3A). Interestingly, the induction is more efficient in the case of *vmlR^E129Q^*. A likely explanation is that at the low antibiotic concentration wild-type VmlR, but not the E129Q variant, efficiently counteracts antibiotic-induced ribosomal stalling, thus suppressing its own induction. Alternatively, the E129Q variant could be more proteolytically stable than the wild-type protein, though there is no mechanistic reason to suspect this to be the case. At 4 μg/ml lincomycin, the wild-type strain can recover growth through expression of wild-type VmlR (Figure 3B), whereas growth and ABCF expression are abrogated in the *vmlR^E129Q^* strain (Figure 3B). Inhibition of growth and ABCF expression of both strains was observed when treated with 40 μg/ml lincomycin (Figure 3C). Collectively, these results indicate that induction of VmlR expression does not require that should be able to efficiently counteract VmlR the very translational defect that triggers its expression.

To determine whether a general defect in translation is able to induce VmlR expression, we took a genetic approach to introduce a global translational defect that does not completely abrogate growth. Deletion of the genes encoding the DExD-box RNA-helicases CshA, CshB and YfmL would cause such a defect in *B. subtilis* by interfering with ribosome assembly, causing cold sensitivity (71); an analogous effect was observed in an *E. coli* strain lacking the DEAD-box helicase SrmB (72). In this case one would expect to observe constitutive induction of VmlR expression in the absence of an antibiotic challenge. The most pronounced effects were observed in the Δ*cshA* strain (VHB224), which exhibited a moderate growth defect at 37°C and a severe defect at 20°C (data not shown). We also characterised the effects of deleting *cshA*, *cshB* and *yfmL* on VmlR expression at 20°C in the absence of antibiotics. While deletion of either *cshB* or *yfmL* had no effect, the expression of VmlR was strongly induced in the Δ*cshA* strain (Figure 3D). In good agreement with previous results (71), the Δ*cshA* strain displayed a clear ribosome assembly defect, as manifested by a pronounced 40S peak and a dramatic imbalance in small and large subunit production, leading to accumulation of free 30S subunits (Figure 3E). While we have not specifically characterised the 40S peak, an analogous 40S peak was observed in Δ*srmB E. coli*, and in this case the 40S particle contained the 23S rRNA precursor associated with an incomplete set of large subunit ribosomal proteins (72). Therefore, it is likely that the 40S particles in Δ*cshA B. subtilis* are also large subunit assembly intermediates, especially given that *B. subtilis* CshA was shown to interact—most likely indirectly—with ribosomal proteins L1 and L3 (71). In contrast, the Δ*cshB* and Δ*yfmL* mutations caused much milder ribosome maturation defects (Supplementary Figure S3). We conclude that significant, but not lethal, perturbation of translation caused by the loss of CshA resulted in a general translational defect that was sufficient to induce VmlR expression without completely abrogating growth. It is important to note that the effect of the loss of CshA is pleiotropic, and it could be that in addition to its role in rRNA processing, the DEAD-box helicase might operate on the highly structured *vmlR* 5′ leader region.

Collectively, our results suggest that induction of VmlR expression is not strictly coupled to the protective function of the ABCF itself, but is instead mediated by the regulatory elements controlling the expression of the *vmlR* ORF. Therefore, we next dissected the roles of the individual regulatory elements located in the *vmlR* leader region.

### NusG-dependent pausing prevents leaky VmlR expression in the absence of antibiotic and potentiates VmlR induction upon lincomycin challenge

**Figure 4.**
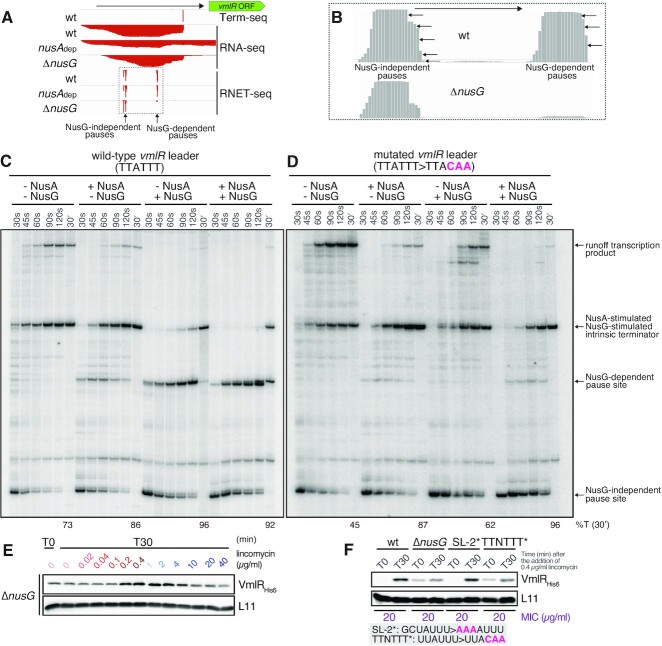
NusG controls lincomycin-dependent induction of VmlR expression. (A, B) Term-seq and RNA-seq (3,47) as well as RNET-seq (3) in wild-type (PLBS730 grown with IPTG), *nusA*_dep_ (PLBS730 grown without IPTG), and Δ*nusG* (PLBS731 grown with IPTG) strains as indicated; ectopic expression of *nusA* is controlled by an IPTG-inducible P_*hy-spank*_promoter. (**A**) The intrinsic terminator in the *vmlR* leader region requires both NusA and NusG for efficient termination. The NusG-dependent and NusG-independent pauses are marked. The arrow at the top indicates the direction of transcription. (**B**) Zoomed in magnification showing the NusG-dependent and NusG-independent pauses. (C, D) *In vitro* reconstitution of NusG-dependent RNAP pausing and NusA/NusG-stimulated termination that controls VmlR expression. *In vitro* transcription termination assays using DNA templates with wild-type (TTATTT, **C**) or mutant (TTACAA, (**D**) NusG pause motifs were performed in the absence or presence of NusA, NusG, or both. Mutations in red are in the consensus NusG-dependent pause motif. Percent termination is shown below each of the 30 min chase reaction lanes. (**E**) VmlR expression is induced by sub-MIC concentrations of lincomycin and the basal expression of VmlR is repressed by NusG. NusG-deficient (Δ*nusG*) strain encoding His_6_-tagged VmlR (VHB489) was treated with increasing concentrations of lincomycin for 30 min (T30) and VmlR_His6_ was detected by α-His_6_ immunoblotting. (**F**) NusG recognizes the TTNTTT motif in the *vmlR* leader region to repress VmlR expression. Wild-type (VHB223), Δ*nusG* (VHB489), SL-2* (VHB612) and TTNTTT* (VHB611) strains were treated with 0.4 μg/ml lincomycin for 30 min (T30) and levels of VmlR_His6_ were assessed by α-His_6_ immunoblotting. Mutations of SL-2* and TTNTTT* are highlighted in red.

We recently mapped NusA-stimulated and NusG-dependent transcriptional pause sites in *B. subtilis* genome-wide by characterising wild-type, NusA-depleted (*nusA*_dep_), and Δ*nusG* strains through sequencing native elongating transcripts followed by RNase I digestion (RNET-seq) (3). We also identified intrinsic terminators that depend on NusA and/or NusG for efficient termination via Term-seq (2,47). These prior studies revealed that the *vmlR* leader contains NusG-independent pause sites near the bottom of SL-1, followed by NusG-dependent pause sites just downstream of SL-2, both of which are upstream of a NusA/NusG-stimulated intrinsic terminator; SL-3 is the terminator hairpin (Figure 1A and Figure 4A, B). Due to the potent stimulation of the *vmlR* leader intrinsic terminator by NusA *in vivo*, *vmlR* is highly overproduced upon NusA depletion (Figure 4A). Taken together, these results suggest important roles of NusA and NusG in controlling VmlR expression.

To further examine the roles of NusA and NusG in the regulation of *vmlR* expression, we conducted *in vitro* transcription assays using a DNA template encoding the WT *vmlR* leader and purified protein components (Figure 4C). By conducting this assay ±NusA and/or ±NusG, we confirmed the existence of NusG-independent and NusG-dependent pausing, as well as the intrinsic terminator. In addition, we observed weak NusA-dependent pausing at the NusG-dependent pause site. To determine whether the conserved NusG-dependent pause motif (Figure 1A, C) functions as predicted (3), we repeated the transcription assay using a template in which the NusG-dependent pause motif had been mutated (TTATTT > TTACAA) (Figure 4D). In this case we observed the NusG-independent and NusA-stimultated pauses, but not the NusG-dependent pause. This template also allowed us to confirm that the intrinsic terminator depends on NusA and NusG for efficient termination; the full-length runoff transcription product was virtually eliminated in the presence of both proteins. Taken together, these results indicate that SL-3 functions as an intrinsic terminator hairpin, that the TTNTTT motif functions as a NusG-dependent pause signal, and that SL-2 likely serves as a NusG-dependent pause hairpin (3).

To probe the role of NusG in VmlR induction *in vivo* we assessed the efficiency of VmlR induction by a lincomycin challenge in the Δ*nusG* (VHB489) strain. The effects of the loss of NusG were two-fold. First, it led to leaky expression of VmlR in the absence of the antibiotic and, second, while the antibiotic stimulates expression, the effect is much weaker compared to the wild-type strain (Figure 4E, compare to Figure 2B). Notably, this level of VmlR expression is still sufficient to confer wild-type levels of lincomycin resistance, with both wild-type and the Δ*nusG* strain having the same MIC of 20 μg/ml. Since the NusG-dependent pause site is located between SL-1 and before the 3′ portion of the AT structure, pausing at this position probably promotes formation of SL-1 (the AAT structure) in the absence of inducing antibiotic. Thus, an increase in AT formation caused by the loss of pausing would explain the leaky expression observed in the absence of NusG. The weaker induction can be explained by the loss of NusG-dependent pausing, which would reduce the time for lincomycin-induced ribosome stalling to occur at the uORF(MIN). In this case, the AAT would form more frequently leading to increased termination.

To further explore the role of NusG-dependent pausing *in vivo*, we constructed two strains predicted to interfere with pausing. One strain (VHB612) contained mutations (SL-2*) that abrogate the formation of SL-2 (GCUAUUU > AAAAUUU), while the other strain (VHB611, TTNTTT*) contained the same mutations in the NusG pause motif that we tested *in vitro* (UUAUUU > UUACAA). SL-2* (VHB612) displayed near-wild-type behaviour upon a lincomycin challenge, both in terms of VmlR expression and lincomycin sensitivity as judged by the MIC (Figure 4F), suggesting that SL-2 is not crucial for NusG-dependent pausing or that this mutation also reduces the ability of the AT to prevent termination, resulting in no net change in expression. However, the TTNTTT* strain phenocopied Δ*nusG* with VmlR expression being leaky in the absence of antibiotic and a lower level of antibiotic-dependent induction (Figure 4F). However, this level of induction is sufficient to provide wild-type levels of resistance as judged by the MIC.

### SL-1 is essential for lincomycin-dependent induction of VmlR expression and SL-3 is essential for repression of VmlR expression in the absence of antibiotics

**Figure 5.**
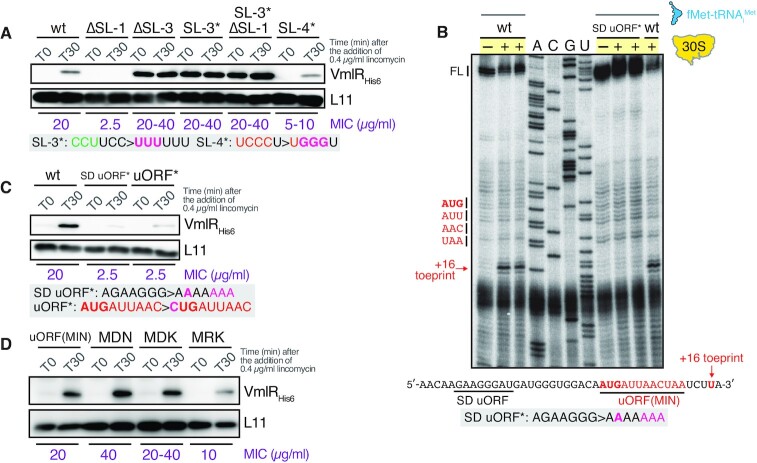
Lincomycin-dependent induction of VmlR expression is caused by ribosomal stalling at the uORF(MIN). (**A**) The effects of truncations/mutations in stem loops SL1, SL3 and SL4 on lincomycin-dependent induction of VmlR expression. SL-1 is essential for lincomycin-dependent induction of VmlR expression and SL-3 is crucial for repression of VmlR expression in the absence of the inducer. Wild-type (VHB570), ΔSL-1 (VHB575), ΔSL-3 (VHB474), SL-3* (VHB572), SL-3* ΔSL-1 (VHB583) and SL-4* (VHB594) cells were treated with lincomycin for 30 min and VmlR_His6_ expression was detected by immunoblotting. Mutation sites of SL-3* and SL-4* are in red. (**B**) 30S ribosomal toeprint of the uORF(MIN). Toeprints were conducted using wild-type (wt) and Shine-Dalgarno sequence mutant (SD uORF*) templates as indicated. Reactions were carried out in the absence (–) or presence (+) of *B. subtilis* 30S ribosomal subunits. All reactions contained *E. coli* fMet-tRNA_i_^fMet^. The start codon (AUG), stop codon (UAA), the position of the 30S ribosomal subunit-dependent toeprint located 16 nucleotides downstream the A residue of the start codon, as well as the full-length reverse transcription product (FL) are marked. Sequencing lanes (A, C, G and U) are shown. (**C**) Translation of the uORF(MIN) is essential for lincomycin-dependent induction of VmlR expression. MIC for lincomycin for wild-type (VHB570), SD uORF* (VHB573) and uORF* (VHB604) strains are indicated in purple. Mutation sites of SD uORF* and uORF* are in red. All experiments were performed at least twice yielding similar results. Expression of VmlR_His6_ was assessed by α-His_6_ immunoblotting. (**D**) uORF sequence variants from other *Bacillus* species are competent in induction of *vmlR* expression in a lincomycin-dependent manner. Wild-type *B. subtilis* uORF(MIN) (VHB570), MDN (VHB839), MDK (VHB840) and MRK (VHB841) uORF variant cells were treated with lincomycin for 30 min and expression of VmlR_His6_ was detected by α-His_6_ immunoblotting.

Next, we either mutationally disrupted (*) or deleted (Δ) stem-loops 1, 3 and 4 and assessed the lincomycin-induced expression of VmlR-His_6_ by immunoblotting as well as the lincomycin sensitivity of the mutant strains (Figure 5A). Deletion of the uORF-containing SL-1 resulted in complete loss of VmlR expression, both in the presence and absence of lincomycin, and sensitised the strain to lincomycin, with the MIC corresponding to that of the Δ*vmlR* strain (2.5 μg/ml). This result strongly supports the importance of the AT, because in the absence of SL-1 there is no way to interfere with formation of the downstream terminator hairpin (Figure 1A). As predicted by our model, both deletion and destabilization (SL-3*, CCUUCC > UUUUUU) of the terminator hairpin (SL-3) resulted in constitutive, lincomycin-insensitive overexpression of VmlR and high levels of lincomycin resistance exceeding that of the wild-type strain (MIC 20–40 μg/ml versus 20 μg/ml). As expected, once intrinsic termination is eliminated, expression of VmlR is no longer dependent on SL-1, since the SL-3* ΔSL-1 (VHB583) strain containing disruptions of both elements displays the same VmlR expression phenotypes as the single SL-3* (VHB572) mutant strain. This result is consistent with SL-1 containing a portion of the AT, which is required to prevent terminator hairpin formation. Mutational disruption of SL-4 (SL-4*, UCCCU > UGGGU) resulted in a moderate defect of VmlR inducible expression and a corresponding increase in lincomycin sensitivity (MIC 5–10 μg/ml), and did not affect the ‘leaky’ expression in the absence of the antibiotic. The latter result is to be expected since in the absence of ribosomal stalling, RNAP naturally terminates upstream of SL-4 (Figure 1A). The near-wild-type functionality of inducible VmlR expression is consistent with the SL-4 sequence not directly participating in the formation of the AT element but rather forming a hairpin what would potentially indirectly promote the formation of the AT element (Figure 1B).

### Translation initiation of the uORF is essential for antibiotic-dependent induction of VmlR expression

Given the crucial role of the uORF-containing SL-1 in control of VmlR expression, we next examined the specific role of the uORF in controlling VmlR expression. Since the VmlR-cognate antibiotics retapamulin and lincomycin stall initiating ribosomes, we performed toeprinting experiments to identify the position of the start codon of the predicted uORF. For this analysis we used *B. subtilis* 30S ribosomal subunits supplemented with aminoacylated and formylated *E. coli* initiator tRNA, fMet-tRNA_i_^fMet^, and either wild-type *vmlR* leader RNA or the same region with mutations in the predicted Shine-Dalgarno (SD) region (SD uORF*, AGAAGGG > AAAAAAA) (Figure 5B). A strong toeprint signal was observed at a position 16 nucleotides downstream of the A in the uORF initiation codon, confirming that this is an authentic initiation codon. Furthermore, the toeprint signal was not observed on the mutant mRNA SD uORF*, thus confirming the predicted location of the SD sequence.

Next, to determine whether translation of the uORF is required for antibiotic-dependent induction of VmlR expression, we constructed *B. subtilis* strains containing the mutant SD region (SD uORF*, VHB573) or a mutant uORF start codon (uORF*, AUGAUUAAC > CUGAUUAAC, VHB604). We assessed the effects of both mutations on VmlR-His_6_ expression levels following a 30-min lincomycin challenge and the lincomycin sensitivity of the mutant strains (Figure 5C). Disruption of the SD sequence and mutation of the start codon virtually eliminated expression of VmlR and phenocopied the Δ*vmlR* strain in terms of lincomycin sensitivity.

Finally, we probed the role of the uORF coding sequence identity in lincomycin-mediated induction of VmlR expression. We have mutated the wild-type *B. subtilis* uORF(MIN) to encode the three sequence variants that are found in other *Bacilli* species: MDN, MDK and MRK (Figure 1C). Both in terms of VmlR induction levels upon lincomycin challenge and the achieved resistance levels (MIC), uORF(MDN) and uORF(MDK) are at least as functional (or even more efficient) as the wild-type *B. subtilis* MIN variant (Figure 5D). The uORF(MRK) variant is less efficient, with the mutant strain being moderately sensitive to lincomycin (MIC 10 μg/ml, a 2-fold decrease) due to significantly compromised efficiency of VmlR_His6_ induction (Figure 5D).

Collectively, our results suggest that the regulatory mechanism controlling VmlR expression is analogous (although probably not homologous) to that described for Streptococcal MsrD macrolide ABCF resistance factor (73). Similar to VmlR, induction of MsrD requires antibiotic-specific (macrolide-specific elongation arrest in this case) ribosomal stalling on the uORF to counter constitutive intrinsic transcription termination. Unlike the case with VmlR, a NusG-dependent pause site was not identified in the *msrD* mRNA 5′ region (73).

### The lack of (p)ppGpp-mediated signalling reduces inducible VmlR-dependent antibiotic resistance

Since incomplete, but significant inhibition of translation is the key to induction of VmlR expression, we reasoned that, similarly to VmlR induction in the translationally compromised Δ*cshA* background, VmlR might be induced in the stationary phase when bacteria experience nutrient limitation and accumulate (p)ppGpp (74). This is due to the fact that (p)ppGpp accumulation greatly reduces translation initiation because (p)ppGpp binds to initiation factor 2 (IF2) (75,76) and to GTPases involved in ribosome assembly (17,77). Furthermore, accumulation of (p)ppGpp induces expression of Hibernation Promoting Factor (HPF) which, in turn, sequesters ribosomes into translationally inactive 100S ribosome dimers (54).

**Figure 6.**
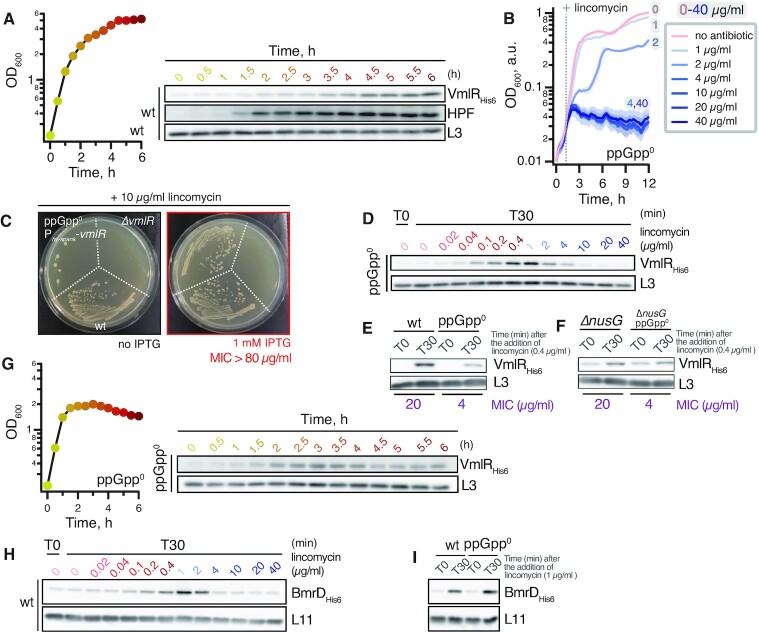
(p)ppGpp is required for full lincomycin-dependent induction of VmlR expression. (**A**) VmlR expression is induced in the stationary phase. Levels of VmlR_His6_ in the time course were assessed by α-His_6_ immunoblotting. Levels of Hibernation Promoting Factor (HPF) were assessed by α-HPF immunoblotting. (**B**) The ppGpp^0^ (VHB237) strain is sensitive to lincomycin. Cells were grown with increasing concentrations (0–40 μg/ml) of lincomycin. Analogous experiments with the ppGpp^0^ strain using low lincomycin concentrations that do not affect bacterial growth (0–0.4 μg/ml) are shown in Supplementary Figure S2B; analogous experiments with Δ*relQ* (NBS1393), Δ*relP* (RIK908) and Δ*relP* Δ*relQ* (NHT436) strains are shown in Supplementary Figure S4. (**C**) Ectopic expression of VmlR rescues the growth defect caused by the presence of lincomycin in the ppGpp^0^ mutant. Wild-type 168 (wt), Δ*vmlR* (VHB5), and ppGpp^0^ P_-*vmlR*_ (VHB452) strains were grown with 10 μg/ml of lincomycin, with or without 1 mM IPTG. In the case of VHB452, *vmlR* expression was under the control of an IPTG-inducible P promoter. (D–F) (p)ppGpp is required for full lincomycin-dependent induction of VmlR expression. (**D**) Lincomycin-dependent induction of VmlR expression in the ppGpp^0^ (VHB237) strain is reduced. VmlR_His6_ levels were assessed after a 30 min treatment with increasing concentrations of antibiotic. (**E**, **F**) Wild-type (VHB223), ppGpp^0^ (Δ*relP* Δ*relQ Δrel*, VHB237), Δ*nusG* (VHB489) and Δ*nusG* ppGpp^0^ (VHB506) cells were treated with lincomycin for 30 min (T30). VmlR_His6_ expression was detected by α-His immunoblotting. MICs of lincomycin for individual strains are indicated in purple. (**G**) VmlR induction in the stationary phase is retained in ppGpp^0^*B. subtilis*. Levels of VmlR_His6_ were assessed by α-His_6_ immunoblotting. (**H**) Treatment with lincomycin induces the expression of BmrD, an ATP-binding protein component of an ABC transport system that confers multidrug resistance. Expression of BmrD_His6_ was monitored by α-His_6_ immunoblotting. Levels of L11 were assessed by α-L11 immunoblotting. (**I**) (p)ppGpp is not important for lincomycin-dependent induction of BmrD expression. Wild-type (VHB622) and ppGpp^0^ (VHB626) strains expressing chromosomally His_6_-tagged BmrD_His6_ were treated with 1 μg/ml lincomycin for 30 min prior to α-His_6_ immunoblotting. All experiments were performed at least twice yielding similar results. Cells were grown on either liquid LB or LB plates at 37°C, expression of both VmlR_His6_ and BmrD_His6_ was assessed by α-His_6_ immunoblotting.

In good agreement with RNA-Seq data showing an increase in *vmlR* mRNA levels upon starvation and stress (78), our immunoblotting experiments show that VmlR-His_6_ protein levels increase in stationary phase, similarly to that of (p)ppGpp-induced HPF (Figure 6A). Knowing that (p)ppGpp accumulation suppresses translation, we next addressed the possible role of (p)ppGpp-mediated signalling in antibiotic-dependent induction of VmlR expression. We found that the *B. subtilis* ppGpp^0^ strain (Δ*relP* Δ*rel*Q Δ*rel*, VHB63) lacking all the RelA-SpoT Homolog (RSH) enzymes responsible for (p)ppGpp synthesis (79) is more sensitive to all of the VmlR-cognate antibiotics but not to linezolid and florfenicol (Table 1, also compare Figure 6B and Figure 2A). To determine whether the higher sensitivity of the ppGpp^0^ strain to VmlR-cognate antibiotics is due to compromised inducible expression of this ABCF and not due to the intrinsically higher sensitivity of the strain, we assessed the lincomycin sensitivity of the ppGpp^0^ strain with VmlR ectopically overexpressed under the control of an IPTG-inducible P_*hy-spank*_ promotor (80) (VHB452). Ectopic expression of VmlR in the ppGpp^0^ background fully restored lincomycin sensitivity (MIC > 80 μg/ml, Figure 6C). We augmented our MIC measurements by directly following the induction of VmlR-His_6_ in ppGpp^0^*B. subtilis*. After a 30-min lincomycin challenge, the VmlR protein levels in the ppGpp^0^ strain were considerably lower than in the wild-type background (compare to Figure 6D to Figure 2B; Figure 6E). In the ppGpp^0^ background, just as in the wild-type strain, expression of VmlR is repressed by NusG in the absence of an antibiotic challenge (Figure 6F). Next, we revisited the induction of VmlR-His_6_ expression upon entry into stationary phase in the ppGpp^0^ strain (Figure 6G). A moderate induction of VmlR expression was still clearly detectable during the transition to stationary phase in the ppGpp^0^ background, suggesting that while important, (p)ppGpp is not essential for VmlR expression upon starvation.

*B. subtilis* encodes three RSH enzymes—Rel, a ‘long’ (i.e. multi-domain) ribosome-associated bifunctional RSH that both synthesises and hydrolyses (p)ppGpp, as well as RelQ and RelP, two monofunctional Small Alarmone Synthetases (SASs) (79). Since SASs are known to be implicated in adaptive stress responses, e.g. upon cell wall-damaging vancomycin and ampicillin treatments (81), we tested whether SASs are also crucial for lincomycin tolerance. All the tested strains (single Δ*relP* (RIK908) and Δ*rel*Q (NBS1393) mutants and a double Δ*relP* Δ*rel*Q (NBS1437) mutant) show wild-type lincomycin sensitivity (Supplementary Figure S4), consistent with the amino acid starvation sensor Rel being the main contributor to (p)ppGpp-mediated lincomycin resistance. Note that the Δ*rel* strain is highly unstable: in the absence of (p)ppGpp hydrolysis that is carried out by Rel, the alarmone produced by the SAS enzymes is not degraded. This results in strong selective pressure yielding suppressor mutants that inactivate the expression of catalytically-competent RelQ and RelP to convert the strain into ppGpp^0^ (82). Therefore, we could not reliably assess lincomycin resistance of the Δ*rel* strain.

Finally, to test whether the observed (p)ppGpp effects are specific for VmlR induction, we characterised the effects of lincomycin on expression of the heterodimeric multidrug exporter BmrC/BmrD in wild-type and ppGpp^0^ backgrounds. Similar to VmlR, expression of this antibiotic resistance determinant is controlled through antibiotic-specific (induced by lincomycin, chloramphenicol and erythromycin, but not kanamycin and gentamycin) transcription attenuation via ribosome stalling on a uORF (26). First, we followed the expression of chromosomally-encoded His_6_-tagged BmrD as a function of lincomycin concentration (Figure 6H). The optimal induction was at 1 μg/ml, which is in good agreement with a previous study where it was reported that maximal induction of GFP expression driven by the native *bmrC* promotor was at 2 μg/ml (26). We then assessed the expression levels of BmrD-His_6_ in the absence or presence of 1 μg/ml lincomycin, both in wild-type and the ppGpp^0^ strains (Figure 6I). In stark contrast to VmlR, we observed equally efficient BmrD induction in the ppGpp^0^ and wild type backgrounds.

Collectively, our results suggest that (p)ppGpp-mediated signalling finetunes inducible VmlR expression upon antibiotic challenge.

## CONCLUSIONS AND PERSPECTIVE

In this study we have dissected a multilayered regulatory mechanism that controls the expression of *B. subtilis* antibiotic resistance factor VmlR. The efficient induction of VmlR upon a ‘cognate’ antibiotic challenge relies on NusG-dependent RNAP pausing, antibiotic-dependent ribosome stalling during translation of an uORF (translation attenuation), transcription attenuation and signaling via the alarmone nucleotide (p)ppGpp.

To our knowledge, VmlR presents the second example of NusG playing an essential role in controlling the inducible expression of an antibiotic resistance determinant, with the first being the *B. subtilis* 23S rRNA methylase TlrB that mediates resistance to macrolide tylosin (25). A dedicated systematic search for NusG TTNTTT pause motifs may uncover the potential involvement of NusG in other mechanisms of inducible antibiotic resistance. Both TlrB and VmlR induction mechanisms rely on antibiotic-specific ribosomal stalling on a uORF. However, they differ in that the induction of TlrB relies on ribosomal arrest by tylosin at translation elongation step at an RYR motif (25), while the induction of VmlR relies on ribosomal arrest during translation initiation. In recent years it has become evident that many protein synthesis inhibitors act context-dependently—i.e. they specifically stall ribosomes translating certain amino acid motifs—and that the molecular mechanisms of context-specific ribosomal stalling shape the amino acid sequences of the regulatory uORF elements that control the expression of resistance determinants (65,66,68,83,84). Therefore, inducible expression of antibiotic resistance determinants driven by antibiotic-induced ribosomal stalling on regulatory uORFs could potentially be defeated by new novel antibiotic variants with altered stalling preferences. By failing to stall the ribosome on the regulatory uORF, these antibiotics would comprise cellular protein synthesis without triggering cellular antibiotic resistance countermeasures. As we show here, when challenged by a novel fully synthetic lincosamide iboxamycin, *B. subtilis* expresses VmlR at a significantly lower level compared with when it is challenged by the natural compound lincomycin. This understanding opens possibilities for further antibiotic design. Finally, as we show here, due to the crucial role of (p)ppGpp in control of VmlR induction, the antibiotic sensitivity pattern of ppGpp^0^*B. subtilis* phenocopies that of antibiotic-sensitive Δ*vmlR* strain (Table 1). This finding further contributes to the appeal of (p)ppGpp-mediated signalling as a drug development target for the attenuation of bacterial pathogens (85).

## DATA AVAILABILITY

The RNET-seq data (3) used in the study are available in the NCBI SRA (BioProject ID PRJNA603835) and GEO (GSE186285), Term-seq (47) data are available in GEO (GSE67058 and GSE 154522).

## Supplementary Material

gkac497_Supplemental_Files
