# Supplementary material for: Expression of Bacillus subtilis ABCF antibiotic resistance factor VmlR is regulated by RNA polymerase pausing, transcription attenuation, translation attenuation and (p)ppGpp
Source: Nucleic Acids Res. 2022 Jun 14;50(11):6174–89. doi: 10.1093/nar/gkac497 (PMC9226507; doi:10.1093/nar/gkac497)
Supplement: gkac497_Supplemental_Files [file gkac497_supplemental_files.zip › Takada_SI.pdf]

SUPPLEMENTARY ONLINE MATERIALS

for

Expression of *Bacillus subtilis* ABCF antibiotic resistance factor VmlR is regulated by RNA polymerase pausing, transcription attenuation, translation attenuation and (p)ppGpp

Hiraku Takada[1,2,3*], Zachary F. Mandell[4], Helen Yakhnin[4], Anastasiya Glazyrina[3], Shinobu Chiba[1], Tatsuaki Kurata[2], Kelvin J.Y. Wu[5], Ben I.C. Tresco[5], Andrew G. Myers[5], Gemma C. Aktinson[2], Paul Babitzke[4,*], Vasili Hauryliuk[2,3,6,*]

[1]Faculty of Life Sciences, Kyoto Sangyo University and Institute for Protein Dynamics, Kamigamo, Motoyama, Kita-ku, Kyoto 603-8555, Japan

[2]Department of Experimental Medical Science, Lund University, 221 00 Lund, Sweden

[3]Department of Molecular Biology, Umeå University, Building 6K, 6L University Hospital Area, 90187 Umeå, Sweden

[4]Department of Biochemistry and Molecular Biology, Center for RNA Molecular Biology, Pennsylvania State University, University Park, Pennsylvania, USA

[5]Department of Chemistry and Chemical Biology, Harvard University, Cambridge, MA, USA

[6]University of Tartu, Institute of Technology, 50411 Tartu, Estonia

[*] to whom correspondence should be addressed:

Hiraku Takada: hiraku.takada@cc.kyoto-su.ac.jp, +81 80 7800 6466

Paul Babitzke: pxb28@psu.edu, +1 814 865 0002

Vasili Hauryliuk: vasili.hauryliuk@med.lu.se, +46 70 60 90 493

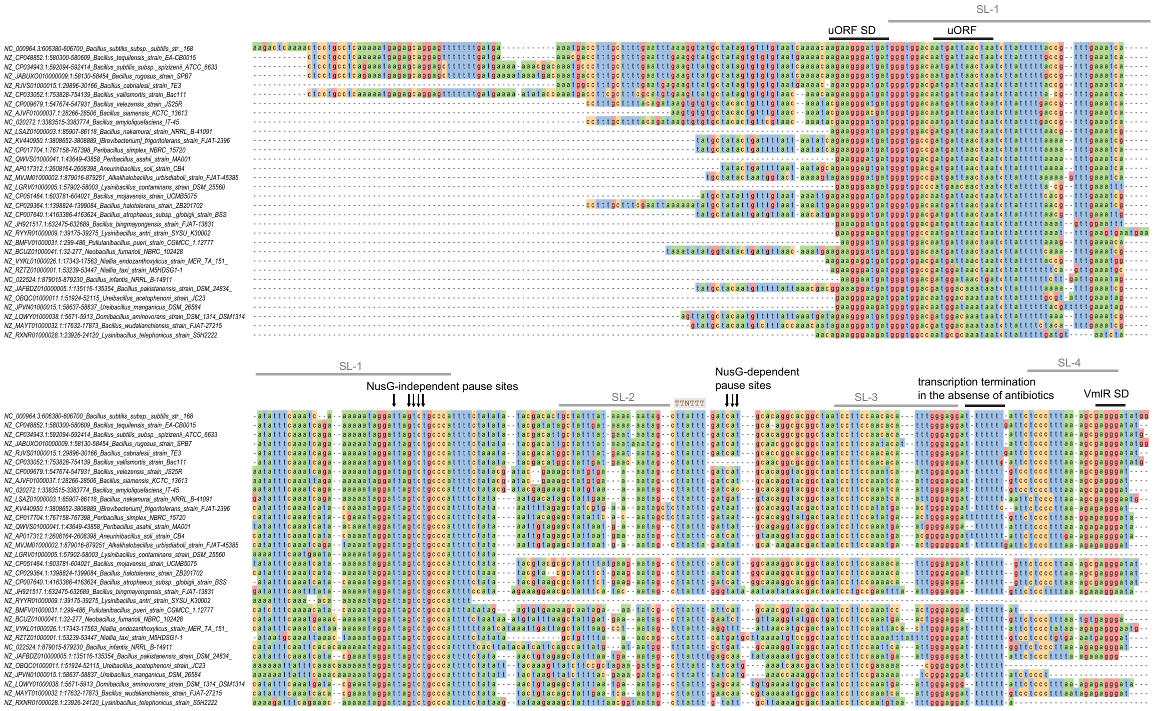

Supplementary Figure 1. Aligned upstream nucleotide regions of *vmlR*, showing conservation of the *vmlR* leader region in various *Bacilli*.

Taxon names are preceded by NCBI nucleotide accession numbers and sequence coordinates. Sequence features are annotated per **Figure 1**.

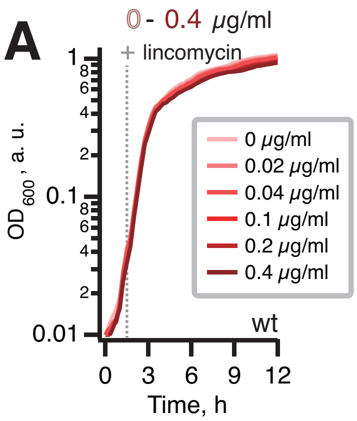

Supplementary Figure 2. Lincomycin does not affect the growth of either wild-type or ppGpp^0 *B. subtilis* at concentrations below 0.4 μg/ml.

Growth of (**A**) the wild-type *B. subtilis vmlR-His$_6$* strain (VHB223) or (**B**) the isogenic *vmlR-His$_6$* ppGpp^0 strain (VHB237) in the presence of 0-0.4 μg/ml of lincomycin. Lincomycin was added once bacterial cultures reached an OD_{600} of ≈0.2. Since liquid media growth experiments were performed in plate format using the Bioscreen C system, the OD_{600} is presented in arbitrary units (a. u.); the standard deviation of three biological replicates is indicated with pale shading. Final concentrations of antibiotics are indicated.

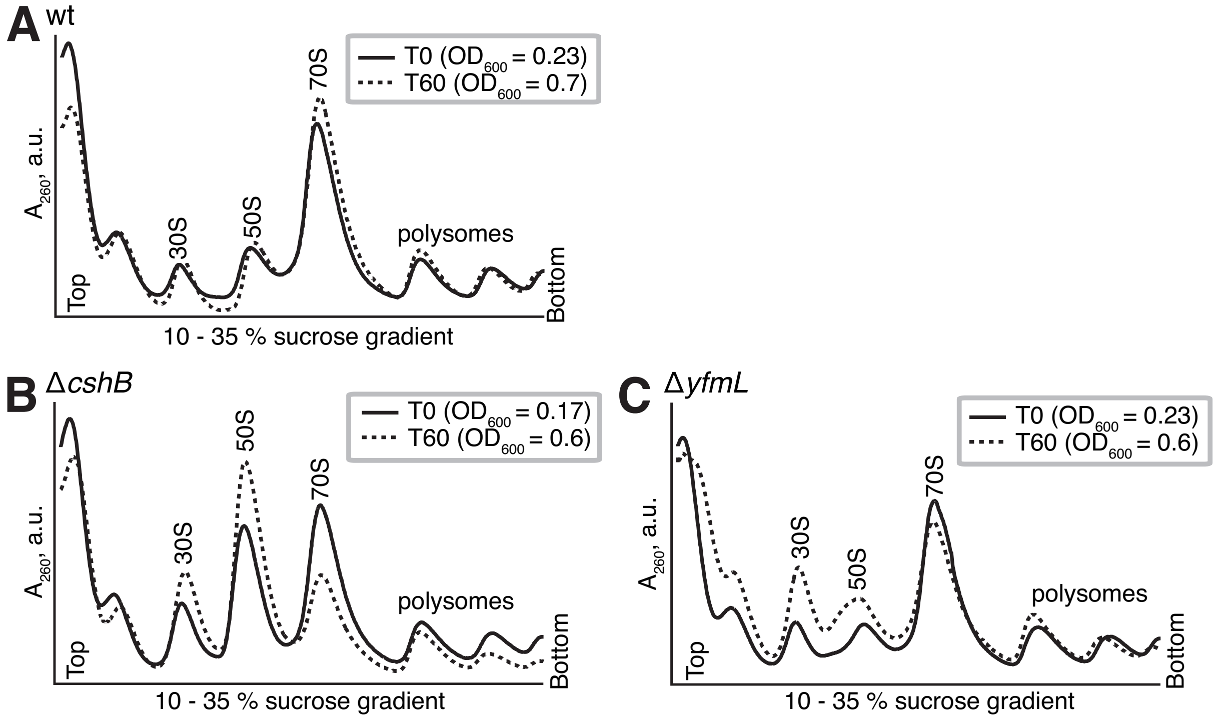

Supplementary Figure 3. Δ*cshB* and Δ*yfmL* mutations cause mild ribosome maturation defects. (**A**-**C**) Polysome profile of wild-type 168 (**A**) as well as isogenic Δ*cshB* (VHB225) (**B**) and Δ*yfmL* (VHB226) (**C**) strains before and after a temperature downshift from 37 °C to 20 °C for 60 min (T60). Experiments were performed at least twice yielding similar results.

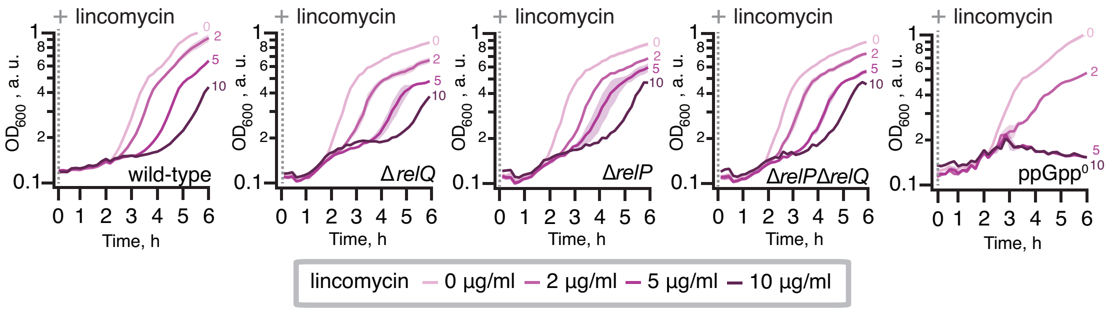

Supplementary Figure 4. RelQ and RelP are not crucial for maintaining growth in the presence of subinhibitory concentrations of lincomycin.

Wild-type 168, Δ*relQ* (NBS1393), Δ*relP* (RIK908), Δ*relP* Δ*relQ* (NHT436) and ppGpp^0 (VHB63) strains were grown with increasing concentrations of lincomycin at 37 °C. Since liquid media growth experiments were performed in plate format using the Bioscreen C system, the OD_{600} is presented in arbitrary units (a. u.); the standard deviation of three biological replicates is indicated with pale shading. Final concentrations of antibiotics are indicated in the figure.
